# Editorial: Integrating Philosophical and Scientific Approaches in Consciousness Research

**DOI:** 10.3389/fpsyg.2021.683860

**Published:** 2021-04-28

**Authors:** Christopher Gutland, Wenjing Cai, Anthony Vincent Fernandez

**Affiliations:** ^1^Department of Philosophy, Center for Documentation and Research of Phenomenology, Sun Yat-sen University, Guangzhou, China; ^2^School of Humanities, Shanghai Jiao Tong University, Shanghai, China; ^3^Department of Philosophy, Kent State University, Kent, OH, United States; ^4^Faculty of Philosophy, University of Oxford, Oxford, United Kingdom

**Keywords:** consciousness research, interdisciplinary, methodology, philosophy, science, phenomenology

Consciousness has a long history as a topic of philosophical investigation. But its status as an object of scientific inquiry is a comparatively recent development. Consciousness seems to elude traditional approaches to scientific research because it is not directly accessible to third-person observational methods or objective systems of measurement. Researchers cope with this challenge in a variety of ways. It is common for scientists to appeal to philosophy to help them think through the nature of consciousness. Philosophical questions might inspire scientific studies and provide theoretical foundations for scientific research programs. And, with the rising interest in interdisciplinary research—including interdisciplinary collaboration—philosophers have likewise developed an interest in how, precisely, they might effectively integrate their conceptual or theoretical contributions with scientific approaches to the study of consciousness. With this Research Topic, we provide a platform for this integration of philosophical and scientific approaches in consciousness research.

Among the questions are: How can philosophical theories of consciousness generate testable hypotheses for scientific research? How should we go about formulating these hypotheses and designing studies that can test them? How can philosophy inform interview techniques that help us better understand the nature of conscious experience? And how can it guide mixed methods that bring together qualitative and quantitative techniques? How can philosophical accounts of consciousness that seem opposed to the assumptions of the natural sciences—such as transcendental theories of consciousness—be made compatible with current scientific research?

This Research Topic brings together a diverse range of articles that address these questions. We briefly introduce each article, proceeding from general methodological reflections to concrete applications and illustrations.

“A Map of Consciousness Studies: Questions and Approaches” by Niikawa is a second order investigation and multidimensional comparison of different theories encountered in consciousness research. It combines a top-down approach, listing and characterizing key questions in consciousness research, with a bottom-up approach regarding the different methodologies being applied. At the end, Niikawa uses the differentiations provided to compare the Integrated Information Theory and the Global Workspace Theory.

Netland, in “The Living Transcendental—An Integrationist View of Naturalized Phenomenology,” draws on Merleau-Ponty's phenomenology to outline a possible integration of the transcendental and the scientific. He characterizes transcendental consciousness as a structure of empirical nature. Through this integrationist view, a dialectical exchange between phenomenology and science can thus be made possible.

In “Ten Testable Properties of Consciousness,” Tyler draws on the philosophy of Emergent Aspect Dualism to present properties of consciousness that are defined phenomenologically, yet testable on the level of neural substrates. Tyler bases this testability on a spatiotemporal isomorphism between the neural substrates and the experiential properties of consciousness, as well as the assumption that, in accordance with his understanding of emergence, the operative principles on the level of consciousness can be functionally dissociated from the levels below it. Tyler then presents 10 such properties and suggests how to test for them.

Keppler and Shani address the relation between the neural correlates of consciousness and its phenomenal, subjective aspect in their article “Cosmopsychism and Consciousness Research: A Fresh View on the Causal Mechanisms Underlying Phenomenal States.” Cosmopsychism assumes the universe is imbued with a ubiquitous field of consciousness that has both an extrinsic physical appearance and an intrinsic manifestation which is phenomenological. The authors argue that when the neural cell assembly coherently oscillates, it acquires phenomenal properties by entering into a temporary liaison with the ubiquitous field of consciousness, whereby the zero-point field in quantum physics is the carrier of both primordial energy and consciousness.

In his article, “Phenomenological Skepticism Reconsidered: A Husserlian Answer to Dennett's Challenge,” Belt reassesses Jean-Michel Roy's notion of phenomenological skepticism. Belt first describes Dennett's arguments against Husserlian phenomenology and presents versions of skepticism that they might imply. The major part of the article reconstructs key features of Husserl's methodology: époche and transcendental reduction, intentional analysis, eidetic variation and intersubjective validation. These reconstructions not only introduce and clarify these methodological facets. They also reveal how Dennett frequently misrepresents them and how they in fact serve to avoid many of the pitfalls Dennett mentions.

In their article, “The Hitchhiker's Guide to Neurophenomenology—The Case of Studying Self Boundaries with Meditators,” Berkovich-Ohana et al. and her team provide a practical guide to Varela's Neurophenomenological Research Program. The authors outline various ways to bridge first- and third-person research using neurophenomenology and argue that researchers should alternate among the bridges depending on their experimental resources and domains of interest. Following this, they provide concrete examples of neurophenomenological studies from their own research program on the dissolution of self-boundaries in meditation.

In “A Phenomenological Paradigm for Empirical Research in Psychiatry and Psychology: Open Questions,” Irarrázaval reflects on the suitability of qualitative research for the phenomenological study of psychopathology. She considers what makes an interview phenomenological, why we should conduct phenomenological interviews with patients, and how to perform an analysis of the data provided by such interviews, among other questions. In closing, she considers the issue of reality's mind-independence within the context of phenomenological research.

The article, “Framing a Phenomenological Mixed Method: From Inspiration to Guidance,” contributed by Martiny et al., aims to integrate qualitative and quantitative methods in a framework of phenomenological mixed methods. By looking into several existing cases that apply mixed methods to the investigation of concrete social phenomena, the article provides guidance on how phenomenology can inform data generation, analysis, and interpretation.

In “The Problem of the Task. Pseudo-Interactivity as an Experimental Paradigm of Phenomenological Psychology,” Wendt stresses the distinction between task and problem while simulating complex social interactions. Reassessing the psychology of thought, Wendt reveals how it offers a means to distinguish between the subject's experiential conceptualization of the task and her motivation to make it her problem. Wendt then introduces pseudo-interactivity, which draws on the psychology of thought and phenomenology, to investigate solely the experiential conditions of the situation in which a subject partakes in a problem-solving task. At the end, Wendt discusses an experiment simulating a pseudo-interactive semantically complex personal interaction.

To counter the denial of a true self, Sparby et al. combine psychology, phenomenology, and narratology in their article “The True Self. Critique, Nature, and Method.” After illustrating the widespread belief in a true self in today's folk psychology and in historical traditions, the authors ask, “Could this belief not be grounded in reality?” Distinguishing between a thin and thick conception of the true self, they offer a defense against the claim that the true self is radically subjective and scientifically unverifiable. Lastly, the authors suggest a method for investigating the true self.

The article, “Inhibited Intentionality: On Possible Self-Understanding in Cases of Weak Agency,” by Ingerslev, studies consciousness in unreflective actions and illuminates an inhibited form of intentionality where weak agency is involved. The article, drawing on phenomenological as well as psychoanalytical insights, proposes a diachronic account of consciousness that can make sense of the possible self-understanding of weak agency in terms of a process of appropriation of one's own action.

Strappini et al., in their contribution, “Empirical Evidence for Intraspecific Multiple Realization?” offer a definition of a psychological property of restored object identification and along this line examine case studies of visually impaired patients to support the Multiple Realization Thesis. This thesis is an anti-reductionist approach claiming both that mental properties are multiply realized and that mental processes can be implemented by different neural correlates.

When considered together, the articles within this Research Topic illustrate how philosophical and scientific approaches can be combined, both in principle and in concrete applications, and how this integration can advance consciousness research.

## Author Contributions

All authors listed have made a substantial, direct and intellectual contribution to the work, and approved it for publication.

## Conflict of Interest

The authors declare that the research was conducted in the absence of any commercial or financial relationships that could be construed as a potential conflict of interest.

